# User Engagement and Usability of Suicide Prevention Apps: Systematic Search in App Stores and Content Analysis

**DOI:** 10.2196/27018

**Published:** 2021-07-14

**Authors:** Chelsey R Wilks, Carol Chu, DongGun Sim, Josh Lovell, Peter Gutierrez, Thomas Joiner, Ronald C Kessler, Matthew K Nock

**Affiliations:** 1 Department of Psychological Sciences University of Missouri-St. Louis St Louis, MO United States; 2 Minneapolis Veterans Affairs Health Care System Minneapolis, MN United States; 3 School of Theology Boston University Boston, MA United States; 4 Department of Psychology Hofstra University Hempstead, NY United States; 5 Rocky Mountain Regional VA Medical Center Denver, CO United States; 6 University of Colorado Anschutz Medical Campus Denver, CO United States; 7 Department of Psychology Florida State University Tallahassee, FL United States; 8 Department of Health Care Policy Harvard Medical School Boston, MA United States; 9 Department of Psychology Harvard University Cambridge, MA United States

**Keywords:** suicide, mHealth, usability, engagement, mobile phone

## Abstract

**Background:**

People with suicidal thoughts are more inclined to seek technology-delivered interventions than in-person forms of treatment, making mobile apps for suicide prevention an ideal platform for treatment delivery. This review examines apps designed for suicide prevention, with a specific focus on user engagement.

**Objective:**

This study aims to update the literature and broadly evaluate the landscape of mobile health apps for suicide prevention; examine apps with key features and primary approaches to suicide prevention; and systematically evaluate the engagement, functionality, aesthetics, and information of the apps.

**Methods:**

All apps related to suicidal thoughts and behaviors were identified in the Google Play and iOS app stores and were systematically reviewed for their content and quality. The mobile app rating scale (MARS) was used to evaluate app usability and engagement.

**Results:**

Of the 66 apps identified, 42 (64%) were specifically designed for people with suicidal ideation, and 59 (89%) had at least one best practice feature for suicide risk reduction. The mean overall MARS score of all apps was 3.5 (range 2.1-4.5), with 83% (55/66) of apps having a minimum acceptability score of 3. The total MARS score was not associated with the user app rating (*r*=−0.001; *P*=.99) or the number of features (*r*=0.24; *P*=.09).

**Conclusions:**

This study identified many usable and engaging apps in app stores designed for suicide prevention. However, there are only limited apps for clinicians. Thus, mobile apps for suicide prevention should be carefully developed and clinically evaluated.

## Introduction

### Background

Suicide is the second leading cause of death in the United States among people between the ages of 10-34 years. Suicidal thoughts and behaviors are difficult to treat, and only a few treatments with evidence of efficacy are widely disseminated. Unfortunately, treatment engagement among suicidal patients is low, particularly among those experiencing frequent and intense suicidal ideation [1-3]. Fortunately, although some high-risk suicidal individuals avoid face-to-face intervention, they may be inclined to anonymously seek out help through technology [4-6].

One cost-effective and convenient avenue for mental health delivery is through mobile mental health apps (ie, mobile health [mHealth] apps). There has been an increase in the number of mHealth app targeted for mental health problems in general [7,8] and suicide in particular [9,10]. mHealth may be a novel strategy to target suicide among those in high-income countries, with over 95% of US adults reporting that they own a smartphone [11]. In addition, approximately 64% of adolescents reported using apps to manage their mental health symptoms [12].

The number of mental health–related apps available to users has increased dramatically, with recent estimates suggesting that more than 10,000 such apps have been created [13]. Unfortunately, only a few mHealth apps have demonstrated efficacy [14,15]. In addition, 74% of users reported that they stopped using mHealth apps after only 10 uses [16]. Thus, there is a significant deficit in studies investigating the efficacy and engagement levels of mHealth apps, as well as those particularly focusing on suicide prevention. For instance, Larsen et al [10] identified 49 apps specifically designed to prevent or reduce suicide and concluded that although many apps contained some elements of best practices, none of the apps provided evidence supporting their efficacy. Best practices for suicide prevention include strategies with consistent evidence for reducing suicide and have been outlined in detail in previous reviews [17]. For example, means safety, defined as the removal of lethal means, has consistently been identified as an effective suicide prevention intervention [18]. Other best practices include providing access to suicide hotlines, crisis planning, and social support [19,20]. De La Torre et al [9] performed a more comprehensive review and identified 20 apps related to suicide prevention and 6 published scholarly articles describing the features and clinical utility of mobile apps for suicide prevention. However, neither of these reviews critically evaluated the user experience of mHealth apps related to suicide prevention. In this context, user experience comprises usability and engagement. Usability refers to how simple and intuitive it is to access computing technology [21], whereas engagement refers to the degree to which user interest is maintained when interacting with computing technology [22]. User engagement can be evaluated using objective metrics (eg, downloads, popularity, and dwell time) and expert ratings [23].

### Objectives

As mHealth apps have the potential to monitor and mitigate suicidal crises, it is important to assess the features and quality of smartphone apps currently available. Apps that can engage users toward more effective coping behavior in lieu of suicidal acts could have a sweeping public health impact but only if the user is prompted to open the app during critical times. Therefore, the objectives of this study were to (1) update the literature and broadly evaluate the landscape of mHealth apps for suicide prevention, (2) examine the key features and primary approaches to suicide prevention of these apps, and (3) systematically evaluate the engagement, functionality, aesthetics, and information of the apps. The systematic evaluation of usability and engagement was difficult until the development of mobile app rating scale (MARS), a tool for classifying and rating the quality of mHealth apps [24]. This review aims to help users make more informed decisions by assessing the features, usability, and engagement of apps designed to prevent suicide.

## Methods

### App Selection

**Figure 1:**
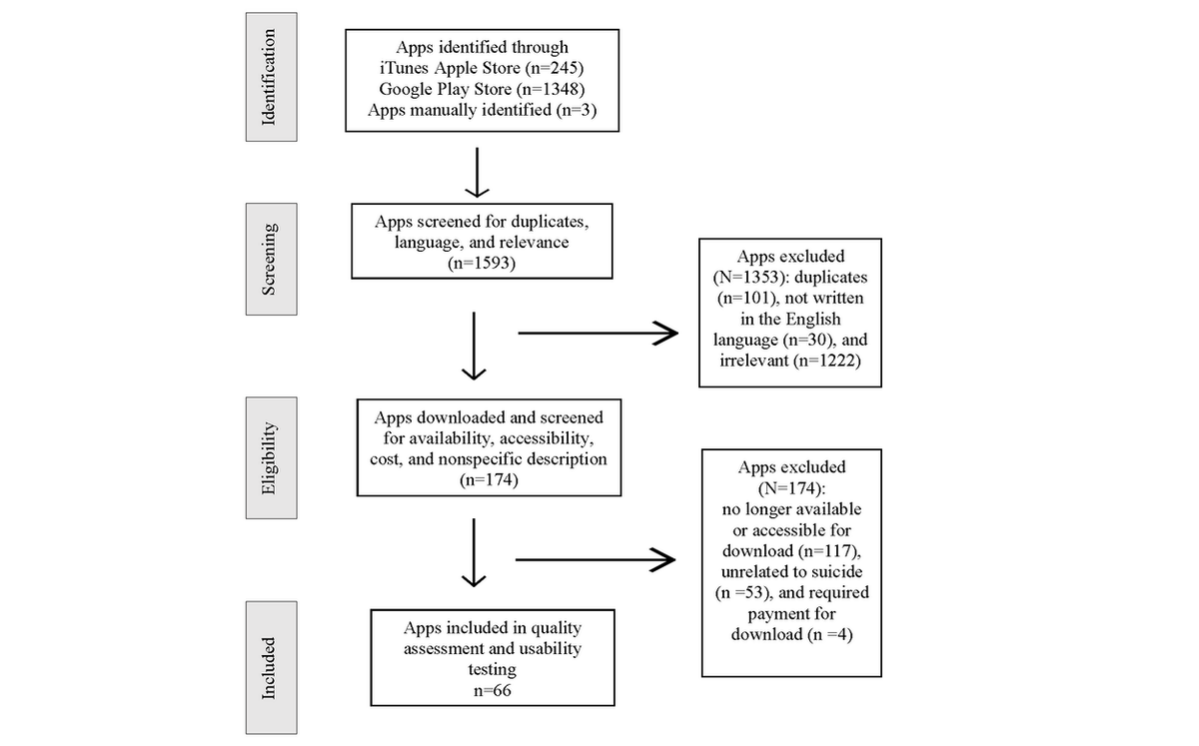
Systematic app selection.

Apps were initially identified in October 2018 and rereviewed in October 2020 through a systematic search of the US iTunes and Google Play stores. Search terms included *suicide, suicidal ideation, suicide ideation, thoughts about suicide, suicidal thinking, ideation, thinking about suicide, self-harm, self-injury, nonsuicidal self-injury*, and *NSSI*. Apps were included if they (1) were smartphone based, (2) used Android or iOS operating systems, (3) were in the English language, (4) had one or more of the aforementioned search terms in the app description, and (5) were available for download in the US app store (iTunes or Google Play). Apps were excluded if they (1) did not primarily target suicidal thoughts, behaviors, or self-injury; required payment for download; or were no longer available or accessible for download (Figure 1). iPhone apps were downloaded and tested using an iPhone 7 and an iPhone 11 in iOS 11, whereas Android apps were downloaded and tested using a simple mobile phone and One Plus 7 Pro Android in version Oreo 8.1 and Oxygen OS 10.3.2, respectively.

### Data Extraction

The following data about all apps were recorded: app name, platform (ie, Android or iOS), current version, cost, number of installs (Android only), and user ratings (1-5 stars). The intended best practice prevention strategy of each app is noted using the relevant portion of the coding scheme by Larsen et al [10], including means safety (ie, reducing access to lethal means of suicide), support (ie, providing access to social support networks, such as through a message board), access to crisis support or helpline, psychotherapies (cognitive behavior therapy or dialectical behavior therapy), and safety planning. Discrepancies were resolved by discussion among the authors until a consensus was reached. As suicide prevention can encompass numerous strategies from support to immediate crisis intervention, we identified four approaches that each app used as its main prevention strategy: providing psychoeducation, teaching coping skills, documenting a crisis plan, and providing social support. Apps that reportedly targeted suicide risk but did not fall into those four categories were classified as *other*.

### App Quality

All apps were rated by 2 independent reviewers using the MARS. The 23 items in the MARS were identified from a review of existing criteria for rating app quality. Each item was rated on a 5-point scale (ie, 1=inadequate; 2=poor; 3=acceptable; 4=good; and 5=excellent) with descriptors provided for each anchor rating. MARS grouped the items into four categories, namely engagement (5 items), functionality (4 items), aesthetics (3 items), and information quality (7 items), and a subjective quality scale (eg, worth recommending and overall satisfaction; 4 items). The dimension of subjective quality in MARS was excluded from the analysis to ensure objectivity and consistency of the assessment process. Previous studies using MARS have also excluded the subjective quality dimension for this reason [24]. The MARS was scored using a mean for each category and an overall mean score. The MARS demonstrated good internal consistency (α=.90) and interrater reliability (intraclass coefficient=0.79) in previous research [24].

Before the app assessment, the 4 reviewers (CRW, CC, DS, JL) discussed the use of the MARS for apps intended for people with suicidal thoughts and behaviors. Evaluating the quality and user experience of mobile apps can be unreliable [25]. The first author (CRW) has extensive research experience in developing mobile apps, conducting user research with mobile and web-based applications, and human-computer interaction. We based the target audience on the following: patients or consumers, clinicians, teens, and family or friends. As recommended by the developers of the MARS, the reviewers considered all items of the MARS and confirmed that all were applicable to apps for suicide prevention and that no additional app-specific items were required.

After a consensus was reached with regard to MARS, the reviewers independently rated the included apps. Each reviewer interacted with the identified app for several minutes, ensuring that all aspects of functionality were tested and evaluated. When reviewers had questions or concerns related to the apps, these issues were discussed among the authors and a consensus was reached.

### Statistical Analyses

Scores were calculated for each MARS item, along with the total mean score. The interrater reliability of the MARS subscales and total quality score was calculated using the intraclass correlation coefficient two-way random-effects model of absolute agreement between single ratings. The mean value for each dimension of MARS was calculated. The difference in app quality between affiliations was analyzed using analysis of variance to examine the moderating effect of developers. Spearman correlations among the four dimensions of MARS, the number of downloads, and average rating were also analyzed. All statistical analyses were performed using SPSS, version 24 (IBM Corporation).

## Results

### Search Results

A total of 1593 apps (iTunes Apple store, n*=*245; Google Play Store, n*=*1348) were initially screened or manually identified. In the screening stage, 1353 apps were excluded as they were either duplicates, not written in the English language, or irrelevant (eg, games, wallpapers, and quotes). Of the 240 apps that were downloaded and tested for eligibility, 174 (72.5%) were excluded as they were no longer available or accessible for download, unrelated to suicide, or required payment for download. The remaining 66 apps were included in the quality assessment and usability evaluation (Figure 1).

### Descriptive Characteristics

The characteristics and the mean MARS scores of the 66 included apps are presented in Multimedia Appendix 1. The average user rating of the apps was 3.5 (range 1-5). Although all included apps were free, 2 offered paid upgraded versions at a cost between US $0.99 and US $149.99 for in-app purchases. A total of 35 apps were found in both iOS and Android app stores, whereas 19 were iOS only and 12 were Android only. More than half (37/66, 56%) of the apps included a privacy policy.

The five features considered to be best practices for suicide prevention were examined for each app and are presented in Table 1. None of the apps had all five features, and only 4 apps had four of the five best practices: *Prevent Suicide: Dumfries & Galloway, ReMinder Suicide Safety Plan, SafetyNet: Your Suicide Prevention App,* and *Don’t Panic–depression and panic help.* The average number of features across apps was 1.7. None of the features was found in the following 7 apps: *SafeUT, R U Suicidal?, Self Harm Recovery, A Teen Suicide Prevention Anime, Seeking the Military Suicide Solution, Elijah,* and *Suicide Prevention-Ways to Help a Suicidal Friend.* The most common of the five features included were access to a crisis line (37/66, 56%), support (33/66, 50%), and a safety plan (22/66, 33%), whereas means safety was the least integrated feature (8/66, 12% of apps). The apps were most often designed for persons experiencing suicidal thoughts (49/66, 74%), followed by friends and family (11/66, 16%), teens (6/66, 9%), and clinicians (1/66, 2%; Multimedia Appendix 1). On the basis of the Android apps alone, the apps with the most downloads were *Calm Harm*, *Talk Life*, and *Mood Tools* with over 100,000 downloads.

**Table 1:** Best practice features for suicide prevention of included apps (N=66).

App name	Means safety	Support	Crisis line access	Treatment	Safety plan
Calm in the Storm: Stress Management					✓^a^
INSIST		✓			
MY3 Support Network			✓		✓
TalkLife for Stress & Anxiety		✓			
SafeUT					
First Step OR		✓	✓		
MoodTools-Depression Aid				✓	✓
HOPE-Broome County Mental Health		✓	✓		
STOPP app		✓		✓	
Jason Foundation A Friend Asks		✓	✓		
Suicide Safety Plan	✓				✓
Safe Students		✓	✓		
Got your back			✓	✓	✓
Stanley-Brown Safety	✓		✓		✓
Operation Reach Out	✓	✓			✓
trustTalk247		✓	✓		
Just in Case for Colleges		✓	✓		
Relief Link			✓		
Ulster County Speak		✓	✓		
Friend2Friend		✓			
be safe suicide safety plan			✓	✓	✓
Every Teen Seen		✓			
distract					✓
Be Safe			✓		✓
Calm Harm-manages self harm				✓	
R U Suicidal?					
Say Something		✓	✓		
Anemone Crisis App			✓	✓	✓
Prevent Suicide-Highland		✓	✓		✓
There is Hope		✓	✓		✓
A.L.E.R.T.			✓		✓
Self Harm Recovery					
Suicide? Help? Tayside					✓
Stay Alive					✓
Dutchess County HELPLINE		✓	✓		
The LifeLine			✓		✓
DMHS^b^: Suicide Prevention Info		✓			
Is S/O Suicidal?		✓			
Did someone you know suicide?		✓			
Step Up and Speak Out		✓	✓		
Kokua Life		✓	✓		✓
MSE&SUICIDE ASSESSr		✓			
Calm Care			✓	✓	
My Shiny Thing				✓	
PMCS Combating Suicide			✓		
SeeSave/See Something Save Someone		✓	✓		
Community Stress First Aid		✓			
iHelp Sunshine Coast	✓	✓	✓		
MS DMH-Shatter in the Silence			✓		
Better Stop Suicide	✓				✓
SCNG^c^ Suicide Prevention		✓			
Alaska Careline		✓	✓		
Prevent Suicide: Dumfries & Galloway		✓	✓	✓	✓
TheHopeLine		✓	✓		
MYPLAN-your safety plan			✓		✓
ReMinder Suicide Safety Plan	✓	✓	✓	✓	
SafetyNet: Your Suicide Prevention App	✓	✓	✓	✓	
TUFMINDS			✓	✓	
UnCut App		✓			
Don’t Panic–depression and panic help	✓		✓	✓	✓
Emotional Support Helpline Directory			✓		
Yellow Ribbon Foundation			✓		
A Teen Suicide Prevention Anime					
Seeking the Military Suicide Solution					
Elijah					
Suicide Prevention-Ways to Help a Suicidal Friend					

^a^Feature present.

^b^DMHS: Durham Mental Health Services.

^c^SCNG: South Carolina National Guard.

### Usability and App Quality

The mean overall MARS score of all apps was 3.5 (range 2.1-4.5), and 83% (55/66) of apps had a minimum acceptability score of 3.0 (Table 2). In general, the *Calm Harm: Manages self harm* app had the highest MARS overall score (4.5), followed by *INSIST* (4.49), *MY3 Support Network* (4.4), and *Talklife for Stress and Anxiety* (4.4). Apps with the highest MARS scores and at least three of five best practice features for suicide prevention were *Got your back, Stanley-Brown Safety*, and *Operation Reach Out*.

Data on the MARS subscale scores and the overall MARS scores categorized according to the main app approaches are presented in Table 2. The interrater reliability was within the acceptable range for each subscale (0.73-0.94), with the highest interrater reliability for information (0.94) and the lowest for function (0.73). Apps with the primary function of providing users with support had the highest overall MARS (mean 3.7, SD 0.8). Coping skills apps scored the highest in engagement (3.2) and function (4.0). In the aesthetics and information subscales, support apps (3.8 and 4.2, respectively) had the highest scores.

**Table 2:** Mobile app rating scores according to main approach (N=66).

Main approach	Count, n (%)	Engagement, mean (SD)	Functionality, mean (SD)	Aesthetics, mean (SD)	Information, mean (SD)	Overall, mean (SD)
Crisis plan	12 (18)	3.13 (0.77)	3.96 (0.59)	3.38 (0.70)	3.64 (1.16)	3.53 (0.76)
Support	24 (36)	2.91 (0.76)	3.86 (0.71)	3.82 (0.72)	4.23 (0.53)	3.70 (0.83)
Psychoeducation	17 (26)	2.56 (0.65)	3.80 (0.54)	3.34 (0.66)	3.70 (1.00)	3.35 (0.87)
Coping skill	10 (15)	3.24 (0.64)	4.00 (0.64)	3.43 (0.83)	3.69 (0.87)	3.59 (0.78)
Other	3 (5)	2.40 (0.35)	3.4 (0.88)	2.50 (0.50)	3.66 (1.44)	2.99 (0.96)

### Correlation Analysis

The total and subscale MARS scores were all significantly correlated, indicating that app quality was consistent across all areas assessed (eg, apps scoring high on engagement also tended to score high on function, aesthetics, and information). The overall MARS score was neither correlated with user app rating (*r*=−0.001; *P*=.99) nor with the number of features included within the app (*P*=.09; Table 3).

**Table 3:** Correlations among total mobile app rating scale (MARS) score, four MARS dimension scores, rating, and number of features.

Characteristics				MARS^a^											Rating			Number of features
				Total		Engagement		Function		Aesthetics		Information						
**MARS**																		
	Total		1.00		—^b^		—		—		—		—			—		
	**Engagement**																	
		Correlation factor	0.72		1.00		—		—		—		—			—		
		*P* value	<.001		—		—		—		—		—			—		
	**Function**																	
		Correlation factor	0.80		0.40		1.00		—		—		—			—		
		*P* value	<.001		<.001		—		—		—		—			—		
	**Aesthetics**																	
		Correlation factor	0.83		0.60		0.50		1.00		—		—			—		
		*P* value	<.001		<.001		<.001		—		—		—			—		
	**Information**																	
		Correlation factor	0.82		0.35		0.63		0.56		1.00		—			—		
		*P* value	<.001		.002		<.001		<.001		—		—			—		
	Rating		−0.01		−0.01		−0.04		−0.01		0.05		1.00			—		
	Number of features		0.20		−0.02		0.22		0.09		0.32^c^		0.24			1.00		

^a^MARS: mobile app rating scale.

^b^Not applicable.

^c^*P*=.006.

## Discussion

### Principal Findings

We examined the user experience, usability, and engagement of mHealth apps designed for suicide prevention. There are three main findings of this study. First, although the majority apps included elements of best practices to reduce suicide risk, none included all these features. Second, most of the reviewed apps were designed for suicidal individuals, rather than for clinicians, friends, and families. Third, the MARS score of the majority of apps was in the *acceptable* range, with the apps designed for support receiving the highest rating; however, the star ratings of users were not correlated with MARS scores, suggesting that star ratings may be indicative of another construct rather than app quality.

Since the app review by Larsen in 2016 [10], nearly twice as many apps designed to reduce suicide have been introduced in app stores. The majority of the apps (59/66, 89%) reviewed in this study have at least one best practice element for suicide prevention. The most common feature across the apps was the inclusion of a crisis line access (37/66, 56%); however, only 12% (8/66) of apps included a means safety feature. Mean safety is considered one of the most potent suicide prevention strategies [26]; thus, it is surprising that it was the least integrated feature. However, integrating content into mobile devices requires intentional design considerations, such as interaction or navigation, which can influence how users learn and engage with the material [27]. In addition, including content on means safety may have been difficult to design and implement. We found no association among the number of best practice elements, MARS scores, or app ratings, indicating that app quality is not solely driven by content, but rather how the content functions and is designed. Notably, an app that implements one aspect well, such as developing a safety plan, may be a better app than one that tries to integrate several features. The apps that yielded the highest MARS scores had a narrow scope. However, this review did not clinically evaluate or evaluate the available research on any of these apps; therefore, it is unclear whether these apps are effective at reducing suicidal crises.

The majority of the apps (49/66, 74%) were specifically designed for suicidal individuals, only 15% (10/66) of which were designed for friends or family and 2% (1/66) for clinicians. This highlights a potential deficit in apps that are designed to treat, manage, or cope with individuals at risk of suicide. A major obstacle in overall suicide prevention is the lack of willingness in treating suicidal individuals among mental health providers [28]. In a previous study, a technology-delivered suicide risk assessment and management tool was associated with reduced fear and increased self-efficacy among clinicians treating suicidal individuals [29]. Given that suicidal people tend to avoid face-to-face treatment [1-3], one avenue to potentially reduce suicide risk could be through a suicide prevention app for friends and family. Although these apps exist in app stores, it is unclear whether they are effective or widely used. Overall, more research is needed to develop and evaluate suicide prevention apps for individuals who work or live with suicidal patients.

Most mobile apps in this review were at least moderately usable and engaging. Although 17% (11/66) of apps yielded unacceptable scores, the average MARS scores were in the acceptable range, indicating that the apps were generally usable and engaging. However, it is unclear whether the apps designed to reduce suicide are reaching the appropriate audience or designed according to what suicidal users need or want. The user context or environment may be a significant driver of the determination of engagement. A suicide app designed to help clinicians assess and manage suicide will likely need to be highly functional, but not necessarily fun to use. In contrast, in apps designed to help users reduce suicidal crises, ongoing app engagement may not be a goal, as app developers hope that suicidal crises will eventually be reduced. This makes the iterative design of suicide-related apps challenging because repeated use may not be an ideal outcome. Traditionally, app developers can use objective measures, such as *time spent on app* and *daily uses*, as outcomes to fine-tune and optimize content delivery; however, these metrics may indicate different factors. An engaging suicide app may be one that is immediately accessible to users during key moments, and previous research on user engagement with mHealth apps indicates that immediate access to resources is an integral aspect that can keep users engaged [30]. As 73% of users tend to stop using a mental health app after 10 uses [16], it is important to understand what factors are associated with discontinued use, such as poor user experience versus *no longer in crisis*. In addition, it may not be profitable to develop a tool to reduce suicide, which may partially explain the relatively large number of nonusable apps designed for suicide. Because developing, publishing, and maintaining mobile apps is time-consuming and expensive, a different funding structure may be required to produce high-quality mobile apps for high-risk users.

In general, there is a lack of research on consumer apps for suicide prevention. Melia et al [31] identified only 7 mobile mental health apps with published outcomes in randomized clinical trials. Although there are apps available to consumers with research support, such as Tec Tec [32] and the Virtual Hope Box [33], these apps were not identified during our search procedure, highlighting a deficit in search term strategy when these apps were released to the public. We were only able to find these apps when we searched for them by name but not when we used our search terms. The function of this review is *user-centered*, as is the case when users search for apps for suicide or self-harm. Researchers who design and develop apps may benefit from the increased marketing of their apps. In addition, users may not be willing to scroll past the first page of results to search for an app, highlighting the importance of the search term strategy and how apps are weighted in the app store search algorithm. Given that star ratings may not accurately reflect app quality, app store algorithms may benefit from another strategy to move quality apps up the list.

### Limitations

Although this paper is the first review to specifically examine the user experience of mobile apps specifically designed for suicide, there are some limitations to the study worth discussing. First, as we only searched on app stores and systematic literature search was not performed, web-based apps that are not featured in app stores were, therefore, not included. Owing to the ease of development and maintenance of web-based apps compared with native apps [25], it is important to include these apps in future research. Second, we only reviewed the user statistics at one time point, and variables such as the number of downloads and user ratings vary over time. Third, as noted previously, some notable and research-supported apps were missing from this study. We believe this illustrates the *lab to marketplace* gap, which is prevalent in all aspects of mental health research but especially in research on dissemination and implementation [34]. Finally, we opted not to evaluate apps that required payment upfront, which limited the scope of our review.

### Conclusions

Although this study identified many usable and engaging apps designed for suicide prevention in app stores, there are several opportunities for mobile app development and enhancement. In particular, there is a lack of apps designed to assist clinicians in treating suicidal patients. In addition, there is a need for more clinical evaluation of suicide prevention apps found in app stores. In general, mobile apps for suicide prevention should be carefully developed and clinically evaluated.

## Appendix

Multimedia Appendix 1 Characteristics and mean mobile app rating scale scores of mobile apps (N=66).

## Abbreviations

MARS: mobile app rating scale
mHealth: mobile health
