# Supplementary material for: User Engagement and Usability of Suicide Prevention Apps: Systematic Search in App Stores and Content Analysis
Source: JMIR Form Res. 2021 Jul 14;5(7):e27018. doi: 10.2196/27018 (PMC8319780; doi:10.2196/27018)
Supplement: Multimedia Appendix 1 [file formative_v5i7e27018_app1.docx]

**Multimedia Appendix 1.** Characteristics and mean mobile app rating scale scores of mobile apps (N=66).

| App name | Overall MARS^a^ | Star rating | | Number of installs^b^ | Version | Cost (US $)^c^ | Platform^d^ | Privacy policy | Developer | Intended audience |
| --- | --- | --- | --- | --- | --- | --- | --- | --- | --- | --- |
|  |  | iOS | Android |  |  |  |  |  |  |  |
|  | | | | | | | | | | |
| Calm Harm-manages self harm^e^ | 4.52 | 4.4 | 4.3 | 500,000+ | 4.3.0 | Free | Both | Yes | Nonprofit | Individuals |
| INSIST^e^ | 4.48 | 3 | N/A^f^ | 100+ | 1.2 | Free | Both | Yes | University | Clinicians |
| MY3 Support Network | 4.4 | 4.3 | 3.7 | 10,000+ | 5.2 | Free | Both | Yes | Government | Individuals |
| TalkLife for Stress & Anxiety | 4.35 | 4.5 | 4.5 | 100,000+ | 6.2.64 | Free^c^ (0.99-149.99) | Both | Yes | University | Individuals |
| SafeUT^e^ | 4.35 | 3 | 3.7 | 10,000+ | 3.2.8 | Free | Both | Yes | University | Individuals |
| First Step OR^e^ | 4.28 | N/A | 5 | 100+ | 1.2 | Free | Android | No | Government | Individuals |
| MoodTools-Depression Aid | 4.23 | 4.8 | 4.3 | 100,000+ | 1.6.5 | Free | Both | Yes | Commercial | Individuals |
| HOPE-Broome County Mental Health^e^ | 4.17 | N/A | N/A | N/A | 1.2 | Free | iOS | No | Government | Individuals |
| STOPP app^e^ | 4.17 | 5 | 4.3 | 10,000+ | 0.0.3 | Free | Both | Yes | Commercial | Individuals |
| Jason Foundation A Friend Asks^e^ | 4.16 | 4.7 | 4.1 | 5000+ | 2.1.0 | Free | Both | No | Nonprofit | Friends and Family |
| Suicide Safety Plan | 4.15 | 4.9 | 3.4 | 10,000+ | 1.8 | Free | Both | No | Commercial | Individuals |
| Safe Students^e^ | 4.12 | N/A | N/A | 10+ | 1.5.6 | Free | Android | No | Commercial | Friends and Family |
| Got your back^e^ | 4.1 | 5 | N/A | N/A | 2.0.2 | Free | iOS | Yes | Nonprofit | Individuals |
| Stanley-Brown Safety | 4.06 | 2.8 | 1 | 500+ | 2.3 | Free | Both | No | Government | Individuals |
| Operation Reach Out | 3.97 | 4 | 4 | 5000+ | 1.0.2.96 | Free | Both | No | Government | Individuals |
| trustTalk247^b^ | 3.97 | 4 | N/A | N/A | 1.2 | Free | iOS | No | Commercial | Individuals |
| Just in Case for Colleges^e^ | 3.94 | N/A | N/A | 500+ | 1 | Free | Both | No | Government | Individuals |
| Relief Link | 3.9 | 3.9 | N/A | N/A | 2 | Free | iOS | No | University | Individuals |
| Ulster County Speak^e^ | 3.89 | 4.5 | 0 | 50+ | 2.4 | Free | Both | Yes | Government | Individuals |
| Friend2Friend^b^ | 3.88 | 4.3 | 0 | 100+ | 9 | Free | Both | Yes | Commercial | Friends and Family |
| be safe suicide safety plan | 3.88 | 5 | N/A | N/A | 1 | Free | iOS | Yes | Commercial | Individuals |
| Every Teen Seen^e^ | 3.87 | 5 | N/A | N/A | 1.2 | Free | iOS | No | Nonprofit | Individuals |
| Distract^e^ | 3.84 | 1 | 3.2 | 5000+ | 1.4.1 | Free | Both | Yes | Commercial | Individuals |
| Be Safe^e^ | 3.84 | 3.4 | 3.7 | 1000+ | 3.6.3 | Free | Both | No | Commercial | Individuals |
| Calm in the Storm: Stress Management^e^ | 3.81 | 4.1 | N/A | N/A | 1 | Free | iOS | No | Government | Individuals |
| R U Suicidal? | 3.81 | 1 | 2.6 | 1000+ | 1.3 | Free | Both | No | Commercial | Individuals |
| Say Something^e^ | 3.77 | 2 | 2.3 | 10,000+ | 4.0.0 | Free | Both | Yes | Nonprofit | Friends and Family |
| Anemone Crisis App^b^ | 3.74 | 4 | N/A | N/A | 1.1 | Free | iOS | Yes | Unknown | Individuals |
| Prevent Suicide-Highland^b^ | 3.69 | 5 | N/A | 500+ | 1.3.0 | Free | Both | Yes | Government | Individuals |
| There is Hope^e^ | 3.68 | 5 | 4.6 | 500+ | 2.1 | Free | Both | Yes | Government | Individuals |
| A.L.E.R.T. | 3.68 | N/A | N/A | N/A | 4.0.04 | Free | Android | Yes | Unknown | Individuals |
| Self Harm Recovery^e^ | 3.62 | 3.9 | 4.4 | 5000+ | 1.0.2 | Free | Both | No | Unknown | Individuals |
| Suicide? Help? Tayside^e^ | 3.61 | 1 | N/A | N/A | 3.3.0 | Free | iOS | Yes | Government | Friends and Family |
| Stay Alive | 3.58 | 4 | 3.8 | 50,000+ | 3.1.0 | Free | Both | Yes | Nonprofit | Individuals |
| Dutchess County HELPLINE^e^ | 3.58 | N/A | N/A | 100+ | 1.3 | Free | Both | Yes | Government | Individuals |
| The LifeLine^e^ | 3.54 | 3 | N/A | N/A | 1 | Free | iOS | Yes | Nonprofit | Individuals |
| DMHS^g^: Suicide Prevention Info | 3.46 | 1.3 | N/A | 500+ | 1.3.1 | Free | Both | No | Government | Individuals |
| Is S/O Suicidal? | 3.45 | N/A | N/A | 100+ | 1.2 | Free | Both | No | Commercial | Friends and Family |
| Did someone you know suicide? ^e^ | 3.44 | N/A | N/A | 100+ | 1.2 | Free | Both | No | Commercial | Friends and Family |
| Step Up and Speak Out | 3.44 | 5 | N/A | 500+ | 2.2.0 | Free | Both | No | University | Individuals |
| Kokua Life^e^ | 3.39 | 5 | 5 | 100+ | 1.1 | Free | Both | No | Government | Individuals |
| MSE&SUICIDE ASSESSr^e^ | 3.36 | 5 | 2.2 | 500+ | 5 | Free (3.99-7.99) | Both | Yes | Government | Friends and/or Family |
| Calm Care^b^ | 3.36 | N/A | N/A | N/A | 1 | Free | iOS | Yes | Commercial | Individuals |
| My Shiny Thing^e^ | 3.29 | 3 | N/A | N/A | 1.4 | Free | iOS | No | Unknown | Individuals |
| PMCS Combating Suicide^e^ | 3.28 | 5 | N/A | 50+ | 19 | Free | Both | Yes | Government | Friends and/or Family |
| SeeSave/See Something Save Someone^e^ | 3.27 | 4.4 | 5 | 1000+ | 3.6.2 | Free | Both | Yes | Unknown | Friends and/or Family |
| Community Stress First Aid^e^ | 3.26 | 5 | N/A | 100+ | 2.2 | Free | Both | No | Nonprofit | Friends and/or Family |
| iHelp Sunshine Coast^e^ | 3.26 | N/A | N/A | N/A | 1.0.4 | Free | iOS | Yes | Nonprofit | Individuals |
| MS DMH-Shatter in the Silence^e^ | 3.24 | N/A | N/A | N/A | 1.0.1 | Free | iOS | Yes | Government | Individuals |
| Better Stop Suicide^e^ | 3.18 | 4.2 | 3.8 | 5000+ | 1.0.37 | Free | Android | Yes | Commercial | Individuals |
| SCNG^h^ Suicide Prevention^e^ | 3.16 | N/A | 5 | 100+ | 1 | Free | Both | Yes | Government | Individuals |
| Alaska Careline^e^ | 3.15 | 5 | N/A | 100+ | 1.2.1 | Free | Both | Yes | Government | Individuals |
| Prevent Suicide: Dumfries & Galloway^e^ | 3.05 | N/A | N/A | 50+ | 1.0.3 | Free | Android | Yes | Nonprofit | Individuals |
| TheHopeLine | 3.03 | 3.4 | 2.9 | 10,000+ | 1 | Free | iOS | N/A | Nonprofit | Individuals |
| MYPLAN-your safety plan^e^ | 3.02 | 2.8 | 3.6 | 1000+ | 3.0.8 | Free | iOS | Yes | University | Individuals |
| ReMinder Suicide Safety Plan^e^ | 2.96 | N/A | 5 | 1000+ | 2.0.1 | Free | Both | Yes | Nonprofit | Individuals |
| SafetyNet: Your Suicide Prevention App | 2.88 | N/A | 2.8 | 1000+ | 1.1 | Free | Android | Yes | Nonprofit | Individuals |
| TUFMINDS^e^ | 2.88 | N/A | 4.6 | 1000+ | 1.0.10 | Free | Android | Yes | Nonprofit | Individuals |
| UnCut App^e^ | 2.86 | 5 | 3.9 | 1000+ | 1 | Free | iOS | N/A | Nonprofit | Individuals |
| Don’t Panic–depression and panic help^e^ | 2.78 | N/A | 4.4 | 50,000+ | 1.53 | Free | Android | Yes | Nonprofit | Individuals |
| Emotional Support Helpline Directory^e^ | 2.62 | N/A | 4.7 | 1000+ | 1.03 | Free | Android | Yes | Government | Individuals |
| Yellow Ribbon Foundation^e^ | 2.35 | 5 | 4.6 | 100+ | 1.3 | Free | iOS | No | Nonprofit | Individuals |
| A Teen Suicide Prevention Anime^e^ | 2.29 | N/A | N/A | 10+ | 2.1.0 | Free | Android | No | Unknown | Individuals |
| Seeking the Military Suicide Solution^e^ | 2.26 | N/A | N/A | 10+ | 1.0.1 | Free | Android | Yes | Government | Individuals |
| Elijah^e^ | 2.25 | 4.3 | N/A | N/A | 1 | Free | iOS | N/A | Unknown | Individuals |
| Suicide Prevention-Ways to Help a Suicidal Friend^e^ | 2.12 | N/A | N/A | 100+ | 2.7 | Free | Android | No | Nonprofit | Friends and Family |

^a^MARS: mobile app rating score.

^b^Installations in Android only.

^c^Upgrade cost.

^d^Available for download in Android or iOS system.

^e^New apps developed and not included in Larsen et al [10] and De La Torre et al [9].

^f^N/A: not applicable.

^g^DMHS: Durham Mental Health Services.

^h^SCNG: South Carolina National Guard.
